# Increased risk of herpes zoster in children with cancer: A nationwide population-based cohort study

**DOI:** 10.1097/MD.0000000000004037

**Published:** 2016-07-29

**Authors:** Hsiao-Chuan Lin, Yu-Hua Chao, Kang-Hsi Wu, Ting-Yu Yen, Yu-Lung Hsu, Tsung-Hsueh Hsieh, Hsiu-Mei Wei, Jhong-Lin Wu, Chih-Hsin Muo, Kao-Pin Hwang, Ching-Tien Peng, Cheng-Chieh Lin, Tsai-Chung Li

**Affiliations:** a School of Medicine, College of Medicine; b Division of Infectious Diseases, Children's Hospital; c Division of Pediatric Hematology-Oncology, Children's Hospital; d Graduate Institute of Biostatistics, College of Management; e School of Post-Baccalaureate Chinese Medicine; f School of Chinese Medicine; g Department of Pediatrics, Chung Shan Medical University Hospital; h School of Medicine, Chung Shan Medical University; i Department of Biotechnology and Bioinformatics; j Department of Healthcare Administration, College of Health Science; k Management Office for Health Data; l Department of Medical Research; m Department of Family Medicine, China Medical University Hospital, Taichung, Taiwan.

**Keywords:** cancer, children, herpes zoster, National Health Insurance program

## Abstract

Herpes zoster is rare in healthy children, but immunocompromised persons have an increased risk of herpes zoster and severe diseases. Considering the very limited information on herpes zoster in children with cancer, we performed a nationwide population-based cohort study to estimate the incidence of herpes zoster in children with cancer and to explore the association between the 2 diseases.

Data were obtained from the National Health Research Institutes Database in Taiwan. A total of 4432 children with newly diagnosed cancer between 2000 and 2007 were identified as the cancer cohort, and 17,653 children without cancer frequency-matched by sex and age at entry were considered the noncancer cohort. The association between herpes zoster and childhood cancer was determined.

Children with cancer had a higher risk of herpes zoster. The incidence rate of herpes zoster was higher in the cancer cohort than in the noncancer cohort (20.7 vs 2.4 per 10,000 person-years; IRR = 8.6; 95% CI = 4.8–15.6). The cumulative incidence was significantly higher in the cancer cohort (*P* < 0.0001). Leukemia, lymphoma, and solid tumor were all associated with the increased risk, and leukemia had the highest magnitude of strength of association.

This nationwide population-based cohort study demonstrated that children with cancer were associated with an increased risk of herpes zoster. In addition to early antiviral treatment, vaccination with heat-treated zoster vaccine or adjuvanted subunit vaccine could be an appropriate policy to decrease the incidence in children with cancer.

## 1 Introduction

Herpes zoster is caused by reactivation of latent varicella-zoster virus (VZV) within the cranial nerve or dorsal root ganglia after primary infection.^[1]^ Herpes zoster is characterized by a vesicular skin rash in the sensory region of the affected ganglia and is often preceded or accompanied by acute pain and itching. Several neurologic complications may develop afterwards, including postherpetic neuralgia, myelitis, cranial nerve palsies, and vasculopathy.^[2]^ Pain, itching, and complications can lead to the impairment in quality of life and even functional disabilities.

Herpes zoster is common in adults with lifetime risk ∼10% to 30% but rare in healthy children.^[3–7]^ Increasing age is the most well-known major risk factor.^[3,4]^ Immunocompromised persons with impaired T-cell immunity are at an increased risk of herpes zoster, such as those with leukemia and lymphoma, recipients of hematopoietic stem cell transplantation, those receiving chemotherapy or immunosuppressants, and those with human immunodeficiency virus infection.^[8–13]^ In children with cancer, herpes zoster can lead to serious complications, including severe post herpetic neuralgia, visceral dissemination, acute or progressive outer retinal necrosis, and even death. The awareness of the symptoms and early treatment are important. However, the association of herpes zoster and childhood cancer is slightly known.

Among studies exploring the incidence of herpes zoster in children with cancer, some had been conducted >2 decades ago,^[14,15]^ and most were conducted in clinical settings of hospitals or health plan.^[4,5,9,16,17]^ Thus, data on herpes zoster in children with cancer should be updated, and nationwide information is especially valuable. In this study, we aim to estimate the incidence of herpes zoster in children with cancer as well as explore the association between the 2 diseases based on the large-scale dataset available from the National Health Insurance (NHI) program in Taiwan.

## 2 Materials and methods

### 2.1 Data sources

This study was approved by the Ethical Review Board of China Medical University (CMU-REC-101-012). The dataset of claims from the National Health Research Institutes Database (NHRID) was obtained from the NHI program in Taiwan, a universal health insurance system implemented by the Ministry of Health and Welfare starting from March 1995 and with a coverage of ∼99% of the 23.74 million residents in Taiwan.^[18]^ The 2000 to 2008 datasets in the NHRID were used, including all claims data of 4432 cancer children aged 18 years or younger identified from registry for catastrophic illness between 2000 and 2007 as well as 17,653 children without cancer randomly selected from beneficiaries being randomly sampled with a selection probability of 0.5 from the entire population of the same age. All selected children were followed up until death, herpes zoster event, withdrawal from NHI, or end of December 2008. The NHIRD contains comprehensive information on patients’ sex, date of birth, details of clinical visits, and details of prescriptions and diagnosed disease codes according to the International Classification of Diseases, Ninth Revision, Clinical Modification (ICD-9-CM). These insurance claims were scrutinized and peer-reviewed by medical reimbursement specialists. The Bureau of NHI performs quarterly expert reviews on random samples of every 50 to 100 ambulatory and inpatient claims in each hospital and clinic to ensure the accuracy of administrative claim data. False diagnostic reports would entail a high penalty. Every individual has a unique personal identification number (PIN). All datasets can be interlinked through individual PINs. Data linked to patient identities were scrambled cryptographically by NHRID to protect privacy.

### 2.2 Study subjects

We conducted a nationwide population-based cohort study that included 2 groups: cancer children and noncancer children. We identified 5128 newly diagnosed cancer children during the period from 2000 to 2007 from outpatient database, and they were recorded in the registry for catastrophic illness. The registry for catastrophic illness database includes data from enrollees who suffer from major diseases and are granted exemption from copayment. The catastrophic illness certification is issued when cancer cases are confirmed by pathological reports. For a patient who meets the criteria of the catastrophic illness database, the attending physician and the medical institution should provide assistance for the application of the catastrophic illness certificate. Afterwards, we excluded those children with incomplete information on sex and residential areas (n = 191). A total of 4432 children with cancer were eligible and their first cancer diagnosis dates were defined as the index date. For the eligible children in noncancer group, an index date between January 1, 2000, and December 31, 2007, was randomly assigned according to the index date distribution of the cancer group. Four controls were frequency-matched by age, sex, residential area, index year, and index month of each case from children without cancer history; thus, a total of 17,653 children without cancer were selected.

### 2.3 Outcome measures and comorbidities

The main outcome event was herpes zoster (ICD-9-CM code 053). Children with herpes zoster should have had at least 3 ambulatory claims or at least 1 inpatient claim with a diagnosis of ICD-9-CM code 053, which was determined by linking records with ambulatory and inpatient care data in the NHRID. The baseline comorbidity history was determined for each patient; these comorbidities included atopic dermatitis (ICD-9-CM code 691.8), allergic rhinitis (ICD-9-CM code 477), or bronchial asthma (ICD-9-CM code 493). Children who had at least 3 ambulatory claims or at least 1 inpatient claim with a diagnosis of one of these ICD-9-CM codes within 1 year of index date were regarded as having comorbidities.

### 2.4 Statistical analysis

Data analysis was performed using SAS version 9.3 (SAS Institute, Cary, NC). The differences in demographics and comorbidities were compared between the 2 groups using the chi-square test for categorical variables and 2-sample *t*-test for continuous variables. Follow-up time was used to estimate the incidence rate of herpes zoster. Follow-up person-years of an individual child was calculated from the index date to the end of December, 2008, the onset of herpes zoster, death, or the date withdrawn from the insurance system. Poisson regression was used to assess the incidence rate ratio (IRR) of herpes zoster and its 95% confidence interval (CI). Cumulative incidence of herpes zoster was computed by the Kaplan–Meier method, and the difference between cohorts was rated by a log-rank test. Cox proportional hazard regression models were used to evaluate the independent effects of herpes zoster by adjusting other variables in the model. Adjusted hazard ratios (HRs) and their 95% CI were estimated. Interactions between herpes zoster and age at entry, sex, urbanization level of residential areas, atopic dermatitis, allergic rhinitis, or bronchial asthma at baseline were examined by adding product terms into the full model, and likelihood ratio tests were used to assess the significance. *P* < 0.05 was considered to be significant.

## 3 Results

A total of 4432 children with newly diagnosed cancers between 2000 and 2007 were identified as the cancer group, and 17,653 children without cancer frequency-matched by sex and age at entry were the noncancer group. The average ages at entry of the cancer group and noncancer group were 8.90 ± 5.68 and 8.91 ± 5.68 years, respectively. The distributions of age at entry, sex, urbanization level of residential areas, and the prevalence of atopic dermatitis were similar in the 2 groups. However, children with cancer had significantly lower prevalence of allergic rhinitis and bronchial asthma (Table 1).

**Table 1:**
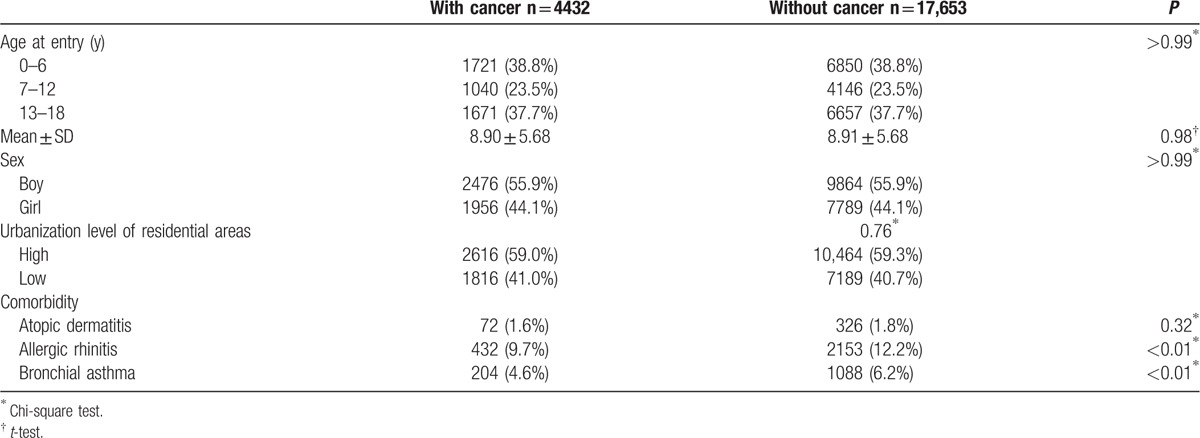
Demographics between children with and without cancer.

**Figure 1:**
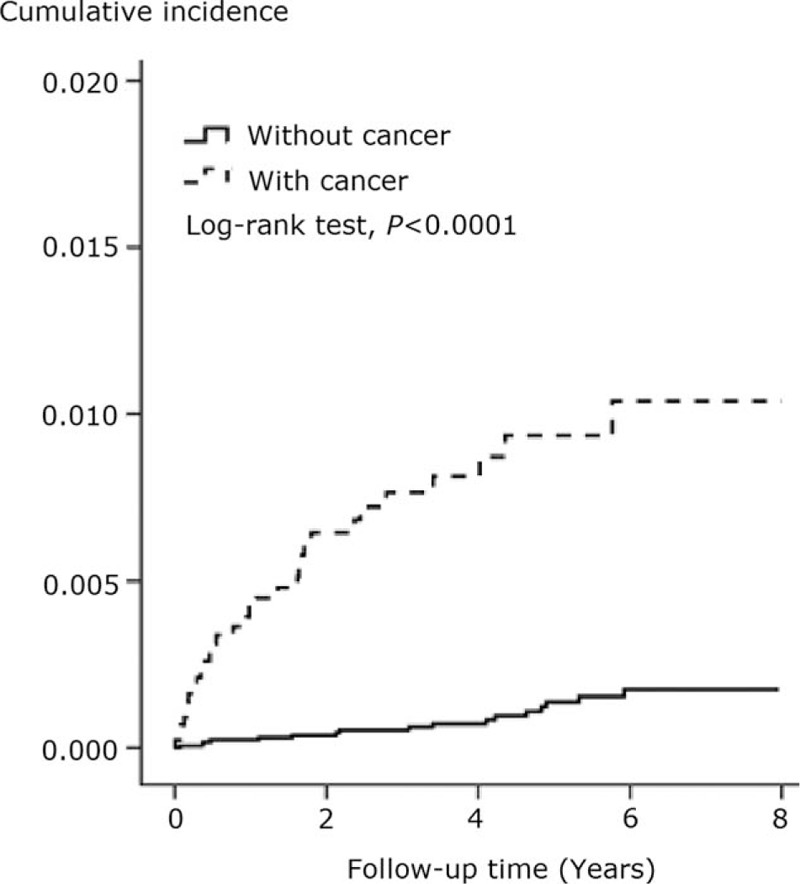
Cumulative incidence of herpes zoster in the study subjects.

As shown in Table 2, the incidence rate of herpes zoster was higher in the cancer cohort than in the noncancer cohort (20.7 vs 2.4 per 10,000 person-years; IRR = 8.6; 95% CI = 4.8–15.6). The incidence rates of herpes zoster in the 2 groups were further stratified based on the age at entry, sex, and urbanization level of residential areas. The incidence rate of herpes zoster was consistently higher in the cancer group. The incidence was the highest in children between 7 and 12 years of age in both groups. The magnitude of IRR was higher in children aged 13 to 18 years at entry (19.6; 95% CI = 4.2–92.1) and in girls (11.0; 95% CI = 4.2–28.6). As shown in Fig. 1, the cumulative incidence of herpes zoster was significantly higher in the cancer group (*P* < 0.0001).

**Table 2:**
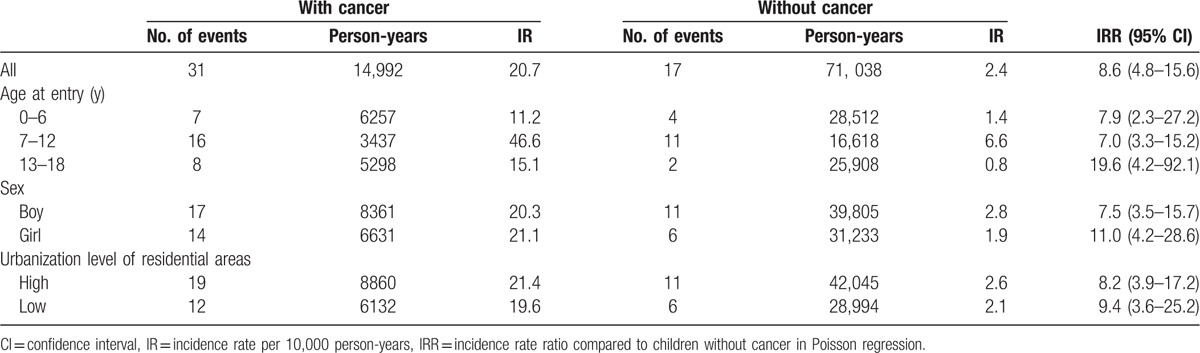
Incidence densities and incidence rate ratios for herpes zoster in children aged <18 years.

Furthermore, children with cancer were categorized into 3 groups according to the cancer types: leukemia, lymphoma, and solid tumor. The multivariate-adjusted HRs demonstrated that leukemia, lymphoma, and solid tumor were all associated with the increased risk of herpes zoster (Table 3). With regard to the strength of association, the magnitude of leukemia was highest, followed by that of lymphoma. Children with leukemia were more likely to develop herpes zoster with the HR of 14.0 (95% CI, 7.0–27.9). The multivariate-adjusted HRs for children with lymphoma and solid tumor were 8.4 (95% CI, 2.8–25.1) and 5.3 (95% CI, 2.5–11.4), respectively.

**Table 3:**
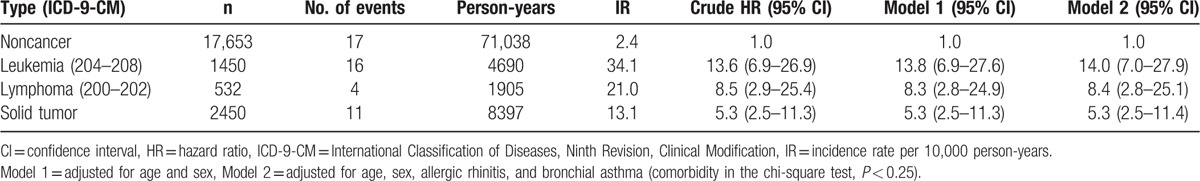
Incidences and hazard ratios for herpes zoster in the study subjects according to the cancer types.

## 4 Discussion

In this first and largest nationwide study, we demonstrated a higher incidence of herpes zoster in children with cancer. The incidence rate of herpes zoster in the population of 4432 children with cancer was 20.7 per 10,000 person-years, and the incidence rate in the population of 17,653 children without cancer was 2.4 per 10,000 person-years. The cumulative incidence was significantly higher in the cancer group (*P* < 0.0001). An increased incidence of herpes zoster has been recognized in children with cancer,^[3–4]^ and >80% of children with lymphoma or acute leukemia developed herpes zoster within 2 years after diagnosis.^[19]^ In 1973, Feldman et al reported that the overall incidence of herpes zoster in a population of 1132 children with cancer was 8.9%, and the incidence was highest (22%) in patients with Hodgkin disease.^[5]^ In 2001, Menon et al reported that herpes zoster was diagnosed in 10 of 188 (5%) children with cancer, and the most common malignancy was leukemia.^[6]^

In this nationwide study, we found that leukemia, lymphoma, and solid tumor were all associated with the increased risk of herpes zoster and hematologic malignancy (i.e., leukemia and lymphoma) had a higher strength of association. The immunocompromised status resulting from intensive chemotherapy could be an important factor associated with the increased risk of herpes zoster. Hematologic malignancy itself may also have additional adverse effects on immunity and may damage the immune functions. In the present study, we found significantly lower prevalence of allergic rhinitis and bronchial asthma in children with cancer. This finding is probably because chemotherapy could impair immune functions and thus decrease the prevalence of diseases from hyperimmunity, such as allergic rhinitis and bronchial asthma.

Cell-mediated immunity is critical to maintain VZV latency. The increased incidence of herpes zoster in bone marrow transplant recipients^[20]^ and lymphoma patients^[21]^ may be related to the weakened VZV-specific cell-mediated immunity. Early antiviral therapy is mandatory for immunocompromised patients. Contrary to live-attenuated vaccine, heat-treated zoster vaccine was safe and immunogenic in immunocompromised adults.^[22]^ Adjuvanted herpes zoster subunit vaccine could reduce the risk of herpes zoster in old adults^[23,24]^ and was also immunogenic and well-tolerated in autologous hematopoietic cell transplant recipients^[25]^ and HIV-infected adults.^[26]^ Therefore, vaccination with heat-treated zoster vaccine or adjuvanted subunit vaccine could be an appropriate policy to decrease the incidence of herpes zoster in children with cancer.

The mechanisms underlying the correlations between herpes zoster and childhood cancer remain unclear. Immunocompromised conditions is a well-known established risk factor for herpes zoster.^[8–13]^ Intense chemotherapy is an important treatment strategy for children with cancer given its capability to provide the best chance of recovery. The extent of compromise in immune functions is generally associated with the intensity of chemotherapy. Sorensen et al found that the risk of herpes zoster is related to the intensity of chemotherapy in children with acute lymphoblastic leukemia,^[7]^ suggesting the possible mechanism of alteration in immune functions in the correlations between herpes zoster and childhood cancer. Moreover, the use of high-dose steroids is part of treatment in children with cancer, especially those with leukemia and lymphoma. Given the multiple effects of steroids in immune functions, we speculated that steroids may attribute to the additional risk of herpes zoster in these patients.

Our study has several advantages. First, it is a nationwide population-based cohort study and comprises the entire claim dataset of children with cancer in Taiwan. Second, children without cancer were randomly selected by frequency matching method from a national dataset that contained half of children population aged 18 years or younger. Third, the population-based study design could facilitate the generalizability of our findings. Finally, the sample size is largest compared with the previous reports.

Our study has some limitations. First, the NHRID does not provide detailed information on patients’ body mass index, level of physical activity, and stage of disease. Second, the diagnosis of herpes zoster was made clinically without confirmation of serology or virology in nearly all patients. However, the clinical diagnosis of herpes zoster is reliable in ∼90% of patients.^[27–28]^ Some patients with herpes simplex might be misdiagnosed as having herpes zoster, and thus, the burden of herpes zoster might be overestimated. However, the biased results from this effect may be toward the null, because the misclassification error was random among children with and without cancer. Third, we categorized childhood cancers only into 3 groups, and no further links to the association of herpes zoster and an individual malignancy were interpreted. Fourth, treatment strategies varied in childhood malignancies, and further links to the association of treatment and herpes zoster were not performed.

## 5 Conclusion

According to the large-scale dataset from NHI in Taiwan, the incidence rates of herpes zoster were 20.7 and 2.4 per 10,000 person-years in children with and without cancer, respectively. Our study found that children with cancer were associated with an increased risk of herpes zoster and leukemia had the highest magnitude of strength of association. We provide useful information about early prevention, diagnosis, and treatment of herpes zoster in children with cancer. In addition to early diagnosis and antiviral treatment, we suggest that vaccination with heat-treated zoster vaccine or adjuvanted subunit vaccine is also needed for children with cancer.
